# 

**Published:** 1909-01-15

**Authors:** 


CONTENTS

Event and Comment - - - - - - - - - 1
Some Suggestions on Artificial Dentures, *By W. A. Giffen, D.D.S.* 5
What Can Oral Prophylaxis and Orthodontia Do For Each Other? *By N. S. Hoff, D. D. S.* 19
Oral Hygiene, *Report of Committee on Oral Hygiene of National Dental Association* - - - - - - - - - 28
Antiseptic Qualities of Saliva, - - - *By Henry C. Ferris* 31
The Main Facts of Dental Jurisprudence. *By Charles S. Ayres, D.D.S.* 37
Is Your Child's Face Beautiful? - *By Henry C. Ferris, D.D.S.* 42
Indents - - - - - - - - - - 46
Announcements - - - - - - - - - - 49

